# Arc Erosion and Wear Induced Particle Emissions in C/Cu Tribo-Pairs of Pantograph–Catenary System

**DOI:** 10.3390/ma19102087

**Published:** 2026-05-15

**Authors:** Wenhao Dai, Pengcheng Cheng, Fulin Mao, Li Xiao, Dehui Ji, Mingxue Shen, Linfeng Min

**Affiliations:** 1 School of Electrical and Automation Engineering, East China Jiaotong University, Nanchang 330013, China; hao.d2005@163.com; 2 School of Materials Science and Engineering, East China Jiaotong University, Nanchang 330013, China; 13795937970@163.com (P.C.); maofulin_2001@163.com (F.M.); sssyxiaoli200217@163.com (L.X.); jidehui_1990@163.com (D.J.); shenmingxue@126.com (M.S.); 3 Rui’an College, Wenzhou Polytechnic, Wenzhou 325035, China

**Keywords:** current-carrying friction, contact load, pantograph–catenary system, carbon contact strip, particulate matter emission

## Abstract

The pantograph–catenary system is a crucial component of rail transit vehicles, performing the vital function of electric energy transmission. During train operation, the current-carrying components continuously emit particulate matter into the surrounding environment due to friction, and these particulate emissions have a significant impact on human health. However, research on the correlation between the current-carrying friction of carbon contact strips and particulate matter emission characteristics is rarely reported. Based on a semi-enclosed pin-on-disc current-carrying friction and wear test rig, this paper investigates the effects of varying current intensity under different contact load conditions on the friction and wear performance of carbon/copper pairs, as well as the associated particulate matter emission behavior. It reveals the damage characteristics of carbon contact strips, the particulate matter emission characteristics, and the relationship between them under different service conditions. The results indicate that the wear mechanism and particulate matter emission behavior of carbon contact strips are jointly influenced by current magnitude and contact load. In the absence of current, increasing the load exacerbates the mechanical wear on the carbon friction pair surface, while elevating the emission concentration of particles of various sizes and stabilizing the particle size distribution. Under current-carrying conditions, a higher contact load effectively reduces the frequency of arc discharges between the friction pair. Meanwhile, the degree of arc erosion on the contact surface worsens with increasing current intensity. Arc discharges instantaneously lead to a sharp increase in particulate emissions, and the higher the discharge intensity or the greater the number of discharges, the higher the particulate concentration around the contact pair.

## 1. Introduction

With the rapid development of modern rail transit, the subway has become the preferred mode of travel for urban residents. However, as most subway platforms are located underground, their relatively enclosed and poorly ventilated environment makes it difficult for airborne pollutants to effectively disperse and be diluted [1]. Multiple environmental monitoring studies have shown that the wear and tear of various components of subway trains during operation is the main source of particulate matter, and the content of metal elements in particulate matter inside subway stations is generally higher than that in outdoor environments [2,3]. Furthermore, chronic exposure to such high concentrations of particulate matter poses potential health risks to the human respiratory system and cardiovascular function [4]. Wang et al. [5] monitored particulate matter emissions in subways and found that the highest average concentrations of PM_2.5_ and PM_10_ on platforms reached 760 μg/m^3^ and 800 μg/m^3^, respectively. Karlsson et al. [6] conducted a comparative genetic study of particulates from subways and streets, revealing that subway particulates have eight times and four times the capacity to cause human DNA damage and oxidative damage to lung cells, respectively, compared with street particulates. Epidemiological studies indicate a positive correlation between particulate matter concentration levels and the incidence of respiratory and cardiopulmonary diseases [7].

To identify the main sources of particulate matter during train operation, researchers have carried out extensive source apportionment work. Shan et al. [8] collected particulates from different locations within subway stations and conducted an analysis on the proportion of their contribution. They found that particulates generated by wheel–rail friction accounted for 29.9%, while the combined contribution of braking and the pantograph–catenary system accounted for 4.3%. Colombi et al. [9] analyzed the elemental composition of the collected particulates and found that copper (Cu) was present, with its mass accounting for 2–3% of the total sample mass. Martins et al. [10] quantitatively analyzed the PM_10_ emission concentration from the operation of pantograph carbon strips and contact wires, estimating particulate emissions in the range of 0.14–0.62 g/km. Furthermore, Daniel et al. [11] found that PM_2.5_ and PM_1_ account for 87% and 72.5%, respectively, of PM_10_ emissions.

As the main power supply component of electric locomotives, the pantograph–catenary system inevitably generates arcs during operation due to the high-frequency dynamic contact impact between the carbon strip and the contact wire [12]. Arc erosion causes severe deterioration of the material surface, and there is a strong correlation among the degree of surface damage, the contact pair temperature, and the arc intensity [13]. Zhang et al. [14,15] found that under high vibration amplitudes, intense arc discharge leads to cracks, material spalling, and ablation pits on the surface of the carbon strips. The instantaneous temperature generated by intense arc discharge can exceed 6000 K [16], resulting in melting and softening of copper materials and gasification of carbon materials. The high temperature caused by arc discharge not only alters the wear mechanism between the contact pairs but also induces sulfidation or oxidation of metallic particles generated at the friction interface [17]. Mei et al. [18] found that the wear mechanisms of pantograph–catenary friction pairs mainly include arc erosion, thermally enhanced wear, and adhesive wear. As the current intensity increases, the wear rate of the carbon strips, the contact surface temperature, and the arc discharge energy all increase. Furthermore, arc discharge causes softening of the contact surface material under shear stress, thereby exacerbating the transfer of copper (Cu) material on the friction surface [19], while a longer arc erosion time further intensifies the wear of the carbon strips [20].

However, the aforementioned studies are all limited to either source apportionment of particulate matter or damage analysis of carbon contact strips, and fail to establish a link between the damage mechanism of carbon strips and the characteristics of particulate matter emissions. Therefore, this paper uses pure carbon strips and copper contact wires as test materials to investigate the damage mechanism of carbon strips and its effects on the concentration, number, morphology, and particle size of particulate matter emissions by varying the current magnitude under different contact loads. Furthermore, it establishes a correlation between the current-carrying friction characteristics of the pantograph–catenary system and the particulate matter emission behavior, aiming to provide a theoretical basis for the optimization design of the pantograph–catenary system in rail transit, as well as for the precise control of pantograph–catenary particulate emissions and the achievement of environmental compliance.

## 2. Experimental Section

### 2.1. Test Equipment and Materials

**Figure 1:**
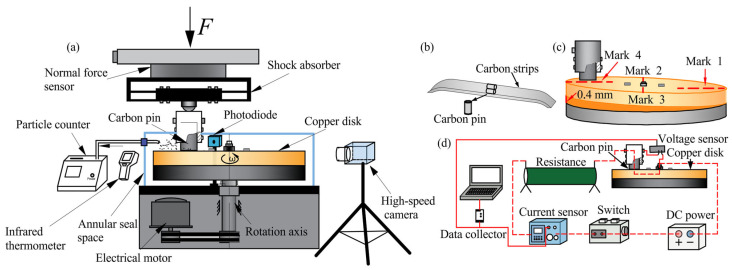
Pin-on-disc current-carrying friction and wear test device: (**a**) schematic diagram of the testing machine; (**b**) carbon pin; (**c**) the unevenness of the copper disc; (**d**) current-carrying test circuit.

The carbon pin used in this test was taken from a pure carbon contact strip currently in service on high-speed railways, which differ moderately from those applied in metro pantographs in terms of actual operating parameters. Nevertheless, considering the research objective of this work on particulate matter emission induced by current intensity under different contact load, as well as the highly consistent material composition between high-speed railway and metro pantograph carbon strips [21], the experimental results still possess great guiding significance for deducing relevant conclusions for metro applications. This material exhibits good mechanical and electrical properties and is widely used in the pantograph–catenary system of electrified railways, with a bulk density of 1.66 g/cm^3^. It was machined into several cylindrical components with a diameter of 10 mm and a length of 30 mm, referred to as carbon pins. The test was conducted on a semi-enclosed pin-on-disc friction and wear tester, with a schematic diagram of the apparatus shown in Figure 1a. In the setup, the carbon pin served as the upper specimen (as shown in Figure 1b), and the copper disc served as the lower specimen. The mating disc was made of red copper with a purity of 99.9% and a radius of 98 mm. The chemical compositions of the two components of the friction pair are listed in Table 1.

The test was conducted on a semi-enclosed pin-on-disc friction and wear tester, with a schematic diagram of the apparatus shown in Figure 1a. In the setup, the carbon pin served as the upper specimen, and the copper disc served as the lower specimen.

The operation between the upper and lower specimens is as follows: the carbon pin is fixed in a holder and maintains vertical contact with the copper disc; a normal load is applied to the carbon pin holder, and the copper disc rotates clockwise. When the power supply is turned on, relative motion between the carbon pin and the copper disc is generated to simulate the sliding contact between a carbon strip and a contact wire. During the test, two-dimensional tangential and normal force sensors are used to detect the tangential and normal forces acting on the contact pair in real time, from which the instantaneous friction coefficient is obtained. The schematic diagram of the current-carrying circuit is shown in Figure 1d. The circuit consists of a DC power supply, a relay, a current sensor, a resistor, the test specimens, and a voltage sensor. The current sensor and voltage sensor record the loop current and the voltage drop across the contact pair in real time, and the data are transmitted to a computer via a data acquisition device, enabling the calculation of the contact resistance of the friction pair. Furthermore, a sealed cover is installed outside the friction pair. Clean air is supplied into the sealed chamber by an air compressor, with an airflow rate set to 2.27 L/min. During the test, a laser dust particle counter (OPC, model CLJ-A3016, Changzhou Sanfeng Instrument Technology Co., Ltd., Changzhou, China) with a sampling frequency of 1 Hz and a recording period of 10 s per cycle is used to monitor the concentrations of particulate matter in six size ranges (0.3, 0.5, 1.0, 3.0, 5.0, and 10.0 μm) per unit volume of air around the friction pair. Meanwhile, the arc intensity is measured using a high-precision, high-speed photodiode with a wavelength response range of 300–1100 nm.

### 2.2. Test Process and Method

To remove the oxide layer from the copper disc surface and achieve good flatness, the disc surface was polished with abrasive sandpapers of 100, 600, 1000, 1200 and 2000 mesh before the test. A dial gauge (Sanliang 312–831, Sanliang Precision Measuring Instrument Co., Ltd., Dongguan, China) with a resolution of 0.0005 mm was used to adjust the copper disc to a horizontal state. Subsequently, pure mechanical friction was applied between the carbon pin and the copper disc until good contact was established (apparent contact area exceeding 80%, indicating the area of visible ploughing on the pin surface), ensuring uniform stress on the carbon pin during the test. The unevenness of the copper disc was then set to 0.4 mm to simulate the periodic unevenness commonly found in contact wires. Based on the degree of unevenness, four positions (1, 2, 3, and 4) were marked on the copper disc, with position 4 being the highest point and position 1 the lowest point, as shown in Figure 1c. The carbon pin was positioned at position 1 of the copper disc, and the test began after verifying that the current circuit functioned normally.

The carbon pin was weighed before and after each test, and the mass loss was taken as the wear amount. The wear rate *w* (g/km) of the carbon pin was obtained by dividing the wear amount by the relative sliding distance of the contact pair. During the test, an infrared thermometer (InfiRay M305, IRay Technology Co., Ltd., Yantai, China) was used every 5 min to measure and record the instantaneous temperature at the contact point between the carbon pin and the copper disc. Furthermore, to reduce the impact of the external environment on the experiments, the temperature and humidity for each experimental group were maintained within a certain range by regulating the laboratory air conditioning and drying equipment. Specifically, the ambient temperature was controlled at 18 ± 2°C, and the relative humidity was kept at 55 ± 5%. The particulate matter generated during the test was divided into two main categories. The first category consisted of inhalable particulate matter emitted in real time into the air around the friction pair. Its particle size and concentration were recorded by a particle counter, and samples were collected by a particle sampler, followed by ultrasonic membrane filtration and drying to obtain the particulates. The second category comprised visible particulate matter (wear debris) remaining on the copper disc after the test, which was collected and preserved following each test run. The test parameters are listed in Table 2. Each test condition was repeated at least three times to ensure the reliability of the results.

A high-definition SAIKEDIGITAL camera (Zoom lens 207150282, Saike Digital Technology Development Co., Ltd., Shenzhen, China) was used to observe the macroscopic wear morphology of the copper disc and the carbon pin. A scanning electron microscope (SEM, HITACHI SU8010, Tokyo, Japan) and an energy-dispersive spectrometer (EDS, X-flash 6I60, Bruker, Billerica, MA, USA) were employed to examine the micro-morphology and analyze the elemental distribution of the worn surface of the carbon pin, the particulate matter collected from the air, and the particulate matter (wear debris) distributed on the copper disc. Meanwhile, a particle size analyzer (PSA 1190, Anton Paar GmbH, Graz, Austria) was employed to measure the particle size distribution of the wear debris collected after the tests.

## 3. Results and Discussion

### 3.1. Current-Carrying Friction Characteristics of Carbon/Copper Friction Pairs

**Figure 2:**
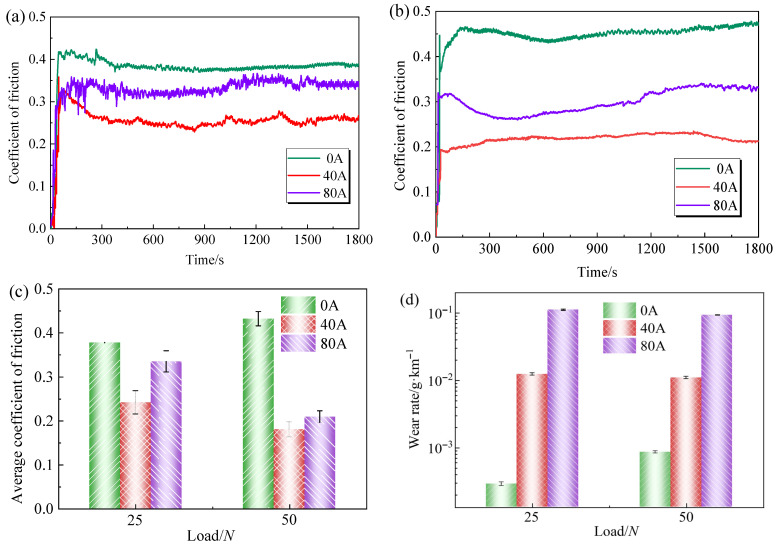
Friction and wear characteristics of the C/Cu contact pair: (**a**) Coefficient of friction under 25 N; (**b**) Coefficient of friction under 50 N; (**c**) Average coefficient of friction; (**d**) Wear rate.

Friction coefficient and wear amount are important indicators for quantitatively characterizing the wear performance of materials. Figure 2 shows the friction coefficient and wear rate of the C/Cu contact pair. As shown in Figure 2a,b, at a contact load of 25 N, the friction curve exhibits the largest fluctuation amplitude at 80 A, followed by 40 A, with the 0 A condition being the most stable. When the load is increased to 50 N, the fluctuation of the friction coefficient is significantly reduced. This reduction is attributed to the fact that after the load increases, the contact becomes more sufficient and stable, and the vibration induced by the unevenness of the copper disc is diminished, thus reducing the fluctuation of the friction coefficient.

As shown in Figure 2c, under different load conditions, the friction coefficient exhibits a U-shaped trend with increasing current, reaching its minimum value at 40 A. Under pure mechanical wear conditions, increasing the load leads to a higher friction coefficient. However, under current-carrying conditions, a higher load results in a lower friction coefficient. From the wear rate (Figure 2d), it can be observed that regardless of the load magnitude, the wear rate of the carbon pin after applying current is significantly higher than that under pure mechanical friction. Moreover, the wear rate at 80 A is more than five times higher than that at 40 A. Under pure mechanical wear conditions, increasing the load aggravates material wear. In contrast, under current-carrying conditions, increasing the load reduces the wear rate.

**Figure 3:**
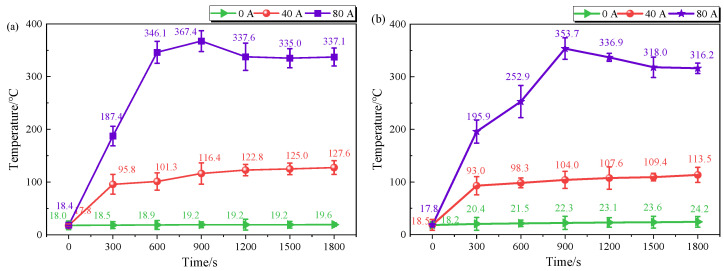
Temperature variation curves of the carbon friction pair under two different contact loads: (**a**) 25 N; (**b**) 50 N.

Figure 3 shows the temperature variation in the friction pair over time under three different current intensities at contact loads of 25 N and 50 N. The heat generated during the current-carrying friction process mainly originates from three sources: frictional heat produced by the relative sliding of contact surfaces, Joule heat generated by electrical resistance as current passes through the contact materials, and arc heat resulting from arc discharge during the friction process [22]. Thus, the temperature of the friction pair rises markedly with increasing current, irrespective of the load. Under non-current-carrying conditions, the friction interface temperature always fluctuates within the range of 20.8 ± 3.4 °C; under this condition, only frictional heat is generated, but it does not cause any notable temperature change at the contact interface. In contrast, under current-carrying conditions, two current levels (40 A and 80 A) were selected: (1) At 40 A, the contact temperature under both loads showed an increasing trend over time. Notably, the temperature at each time point under a load of 25 N was higher than that under a load of 50 N. (2) When the current increased to 80 A, the contact temperature under both loads first increased, then decreased, and finally stabilized. In particular, the temperature under both loads reached its maximum at 900 s. During the early stage of the friction process (300–900 s), the temperature of the friction pair increased significantly, with a rise of more than 150 °C. This sudden temperature increase intensifies the oxidation of the material surface [23], leading to the continuous formation of an oxide film during friction, which serves as a lubricant. This is one of the reasons for the decrease in the friction coefficient during this period, consistent with the variation trend mentioned above.

**Figure 4:**
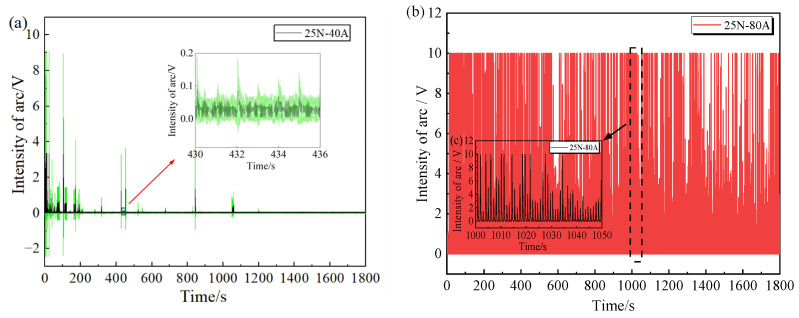
Distribution of arc discharge intensity across the contact pair: (**a**) 40 A; (**b**) 80 A; (**c**) Local magnification at 80 A.

Contact load plays a significant role in regulating arc discharge. At a contact load of 50 N, the contact interface of the friction pair remained stably engaged throughout the test, and no arc discharge was detected. In contrast, at a contact load of 25 N, a dynamic separation gap was formed during the cyclic motion of the carbon pin sliding from position 4 (the highest point) to position 1 (the lowest point) on the copper disc. When the voltage across the contact pair exceeded the breakdown voltage of the air gap, molecules in the air were ionized into plasma, generating an arc [24]. The distribution of arc intensity is shown in Figure 4.

Under 40 A, arcs were mainly concentrated in the early stage of friction (the first 900 s), where only a few high-intensity arcs (6–10 V) occurred, and most arcs were of medium or low intensity (Figure 4a). When the current increased to 80 A, arcs occurred throughout the entire friction process, and high-intensity arcs appeared very frequently (Figure 4b). A local magnification of the arc intensity during the middle stage (1000–1050 s) is shown in Figure 4c. It reveals that under 80 A, the arc intensity was mainly distributed in the range of 4–10, far exceeding the intensity and density of arcs under 40 A. Thus, when conditions for arc generation are present, the current intensity has a substantial influence on the arc intensity.

**Figure 5:**
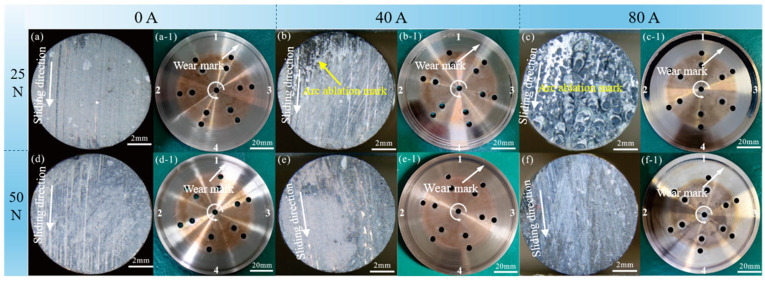
Macroscopic morphology images of carbon pin and copper disc wear surfaces: (**a**,**a-1**) 25 N-0 A; (**b**,**b-1**) 25 N-40 A; (**c**,**c-1**) 25 N-80 A; (**d**,**d-1**) 50 N-0 A; (**e**,**e-1**) 50 N-40 A; (**f**,**f-1**) 50 N-80 A. Note: Numbers 1–4 on the copper disk correspond to the markings at Positions 1–4 in Figure 1c.

To visually observe the effect of arc erosion on the macroscopic morphology of the materials, the post-test material surfaces were photographed, as shown in Figure 5.

Under different operating conditions, the wear tracks on the carbon pin and copper disk surfaces exhibit significant differences. Under no-current conditions, only ploughing is present on the surface of the carbon pin at 25 N (Figure 5a); at 50 N, the ploughing becomes more pronounced (Figure 5d). This is attributed to the increased load, which intensifies abrasive wear. Meanwhile, only shallow wear marks are observed on the copper disk surfaces under both loads (Figure 5a-1,d-1). Under the conditions of 25 N and 40 A, partial arc ablation marks (loose, rough black ablation areas) appear at the exit side of the carbon pin along the sliding direction, and local black wear marks are observed on the copper disk surface, as shown in Figure 5b,b-1. These black marks are formed by wear debris adhering to the copper disk surface. When the current further increases to 80 A, the carbon pin surface becomes almost entirely covered by arc ablation craters and replaced the original abrasion morphology. At the same time, black wear marks on the copper disk account for more than half of the total track area (Figure 5c,c-1), indicating the occurrence of extremely intense arcing in this region (Position 3-2), which causes severe ablation damage to the material and increases debris generation. Under high-load conditions, the surfaces of the carbon pin and copper disk predominantly exhibit mechanical wear marks (Figure 5e,e-1). At 80 A, the ploughing on the carbon pin surface becomes significantly deeper (Figure 5f,f-1).

**Figure 6:**
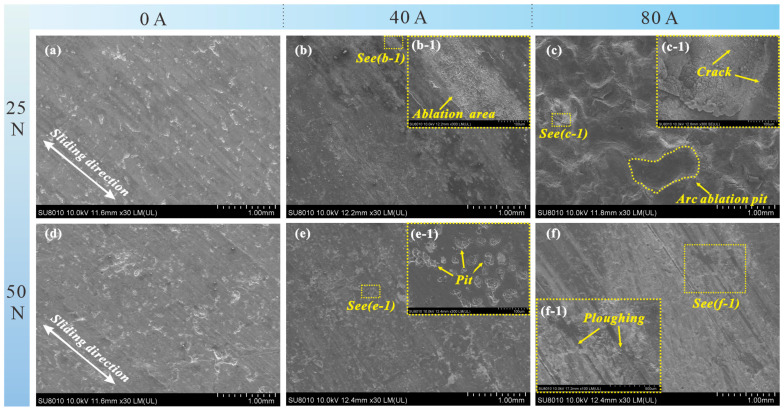
Worn surface micro-morphology of the carbon contact pair: (**a**) 25 N-0 A; (**b**) 25 N-40 A; (**c**) 50 N-80 A; (**d**) 50 N-0 A; (**e**) 50 N-40 A; (**f**) 50 N-80 A. (**b-1,c-1,e-1,f-1**) are enlarged views of the corresponding positions in (**b,c,e,f**), respectively.

Figure 6 shows the surface micro-morphology of the carbon pin under two load conditions with different currents. Under low-load conditions, both the distribution range and the size of individual ablation craters increase significantly with increasing current. At 0 A, the carbon pin surface is dominated by grooves and a few spalling pits (Figure 6a). At 40 A, an arc ablation region (a white band distributed along the sliding direction) appears on the worn surface in addition to mechanical wear marks (Figure 6b). This region is filled with loosely structured, small network-like ablation craters (Figure 6b-1). When the current further increases to 80 A, the worn surface of the carbon pin is severely eroded by arcs; large ablation craters merge into patches, making the entire worn surface uneven (Figure 6c). Magnified observation of the ablation craters reveals delamination and cracks at the crater bottom. The intense temperature difference during arc discharge causes carbonization and even sublimation of the carbon material, leading to the peeling of the surface layer. Meanwhile, numerous nodular structures formed by high-temperature ablation appear at the crater bottom (Figure 6c-1).

Under high-load conditions, the damage caused by current to the material is significantly reduced, and the wear mechanism under all current levels is dominated by mechanical wear. At 0 A, the number and size of spalling pits on the worn surface under 50 N are larger than those under 25 N (Figure 6d). At 40 A, the number of spalling pits on the surface increases notably (Figure 6e). A comparison of Figure 6b-1,e-1 shows that the pits formed by electrical wear and mechanical wear exhibit different morphological characteristics: the arc-eroded surface shows a continuous banded zone, within which the ablation craters appear as loose networks and are mostly circular or elliptical with smooth edges; in contrast, the spalling pits caused by mechanical wear are scattered and mostly irregular in shape. At 80 A, the ploughing on the worn surface of the carbon pin is further deepened (Figure 6f-1), and the white transfer film becomes more extensive (Figure 6f).

### 3.2. Particulate Emission Characteristics of Carbon/Copper Friction Pair

**Figure 7:**
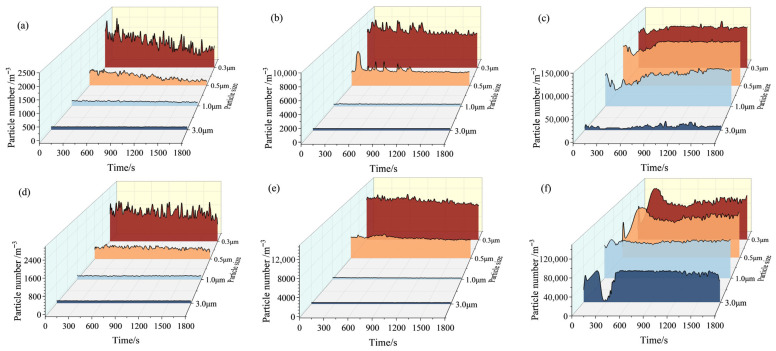
Emission distribution curve of particle number with friction time under different operating conditions: (**a**) 25 N-0 A; (**b**) 25 N-40 A; (**c**) 25 N-80 A; (**d**) 50 N-0 A; (**e**) 50 N-40 A; (**f**) 50 N-80 A.

The particle size detected by the particle counter (category 1 particles) is divided into 0.3, 0.5, 1, 3, 5, and 10 μm. Due to the fact that large-sized particles (5 μm, 10 μm) are produced in very little quantities, there is a difference of three orders of magnitude or more compared to the number of small-sized particles (0.3 μm, 0.5 μm). Therefore, only the number of medium to large-sized particles (1~10 μm) is compared here to illustrate the variation in the number of particles generated by contact pair friction over time, as shown in Figure 7. Under non-current-carrying conditions, the particulate matter emission concentration shows a slow decreasing trend with the progress of the cycle under different contact loads (Figure 7a,d). After the application of current, the emission characteristics became more complex. At a contact load of 25 N and a current of 40 A, the particulate emission concentration fluctuated during the early stage of friction and then gradually stabilized (Figure 7b). Specifically, within the first 900 s, especially for small-sized particles, sharp increases in emission occurred frequently. Combined with the arc intensity distribution under this condition (Figure 4a), it is evident that this phenomenon nearly coincides with the rise in arc discharge intensity. That is, the huge energy released instantaneously by arc discharge causes rapid vaporization and melting of the contact surface material [25], generating a large number of particles that are emitted into the surrounding environment. Therefore, fluctuations in arc intensity directly lead to dramatic changes in the number of emitted particles. In contrast, at a contact load of 50 N (Figure 7e), the emission concentration curve remained stable without significant fluctuations. Under this condition, the carbon pin and the copper disc achieved close contact, effectively suppressing arc discharge and thus greatly reducing particulate emissions.

When the current increased to 80 A at a contact load of 25 N (Figure 7c), the fluctuation amplitude of particulate emissions was relatively large during the initial stage of the test (i.e., the running-in stage [26]). As Joule heat continuously accumulates and electric arcs are generated, particle emissions show a relatively increasing trend and remain at a high level. When the contact load increased to 50 N (Figure 7f), the emission of small-sized particles was relatively high during the early friction stage. Over time, the particulate emissions gradually decreased and eventually stabilized at a low level. The main reason is that under a higher contact load, the cutting force at the friction interface increased, Severe mechanical delamination promotes the emission of small and medium-sized particles. Moreover, although no large arcs occurred during the test, the Joule heat generated by the high current and the frictional heat generated by the interaction of the contact pair caused the initial temperature of the friction surface to rise rapidly to 195 °C. Under such high-temperature conditions, the chemical reactivity of the friction material surface was significantly enhanced, making it more prone to oxidation [27]. During friction, the oxidation products adhered to the wear debris, gradually forming a thick tribofilm at the friction interface. This reduced direct wear on the material surface, causing the number of particles emitted by the material to decrease compared with the initial stage.

**Figure 8:**
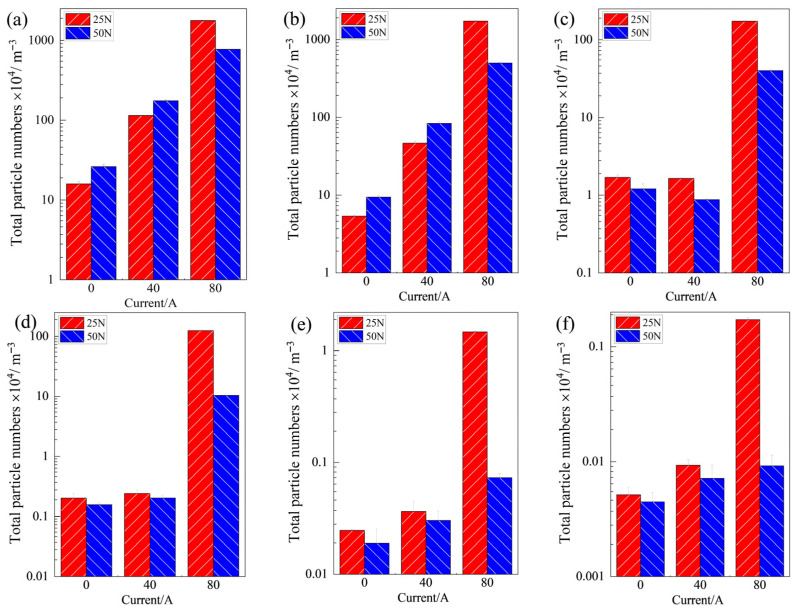
Total emissions of particles of various sizes under different operating conditions: (**a**) 0.3 μm; (**b**) 0.5 μm; (**c**) 1.0 μm; (**d**) 3.0 μm; (**e**) 5.0 μm; (**f**) 10.0 μm.

Figure 8 shows the total number of particulate matter (category 1 particles) of different sizes emitted by the contact pair during the entire friction process under various operating conditions. Overall, the emitted particles were mainly concentrated in the size ranges of 0.3 μm and 0.5 μm. Under the same contact load, as the current intensity increased from 0 A to 80 A, the number of particles in each size range showed a very significant increasing trend, indicating that the increase in current intensity has a direct and strong promoting effect on particulate emissions.

Under no-current and low-current (40 A) conditions, at a contact load of 50 N, the total number of ultrafine particles (smaller than 0.5 μm) emitted was higher than that at 25 N. Conversely, the trend for particles larger than 1.0 μm was opposite. This is because under continuous friction, the higher load of 50 N leads to more thorough grinding of the wear debris on the copper disc surface, causing more fine particles to become airborne and to be released into the surrounding air. When the current increased to 80 A, the total number of particles in all size ranges reached high levels. In particular, at 25 N, the total number of particles in each size range was approximately four times that at 50 N. This is attributed to the fact that at a contact load of 50 N, the contact area between the friction pair increases, thereby reducing the current density on the surface asperities. Meanwhile, the heat generated by the 80 A current at the friction interface can be distributed and transferred more evenly, leading to a relatively milder degree of material degradation.

**Figure 9:**
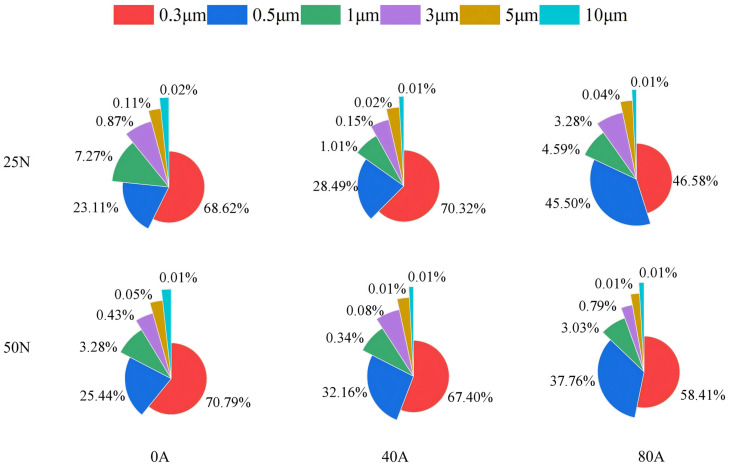
The proportions of particulate matter in six particle sizes emitted under different operating conditions.

To further investigate the effects of different contact loads and current intensities on the particle size distribution of particulate matter emitted during the entire friction process, the proportions of particles (category 1 particles) in six size ranges were statistically analyzed, and the results are shown in Figure 9. It is evident that particles with a size ≤0.5 μm accounted for more than 70% of the total particulate matter in all cases. Under no-current conditions, the proportions of particles larger than 1.0 μm at contact loads of 25 N and 50 N were 7% and 3%, respectively. At a current intensity of 40 A, the vast majority of particles emitted into the air during friction were fine particles (≤0.5 μm), accounting for as much as 99%. When the current intensity increased to 80 A, the proportion of large particles (≥1 μm) under low load reached 26%, whereas at 50 N it increased by only 4%. Thus, an increase in current intensity leads to a higher proportion of larger particles, and increasing the contact load can effectively reduce the proportion of large particles.

**Figure 10:**
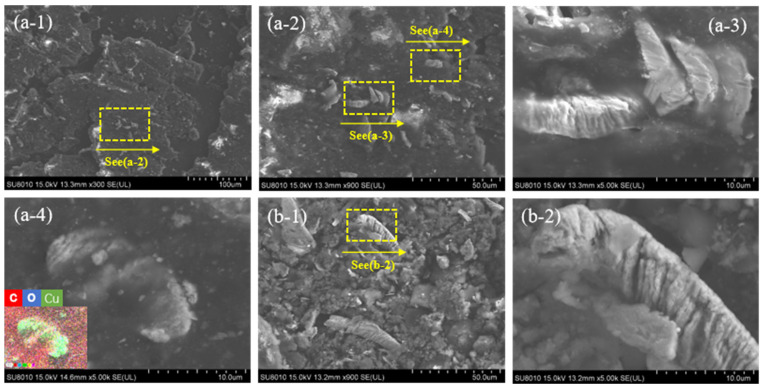
Morphology of wear debris generated by the contact pair under 40 A working condition: (**a-1,a-2,a-3,a-4**) 25 N; (**b-1,b-2**) 50 N.

It can be seen from Figure 10 that the wear debris (category 2 particles) generated by the friction pair at a current intensity of 40 A during the test has a specific morphology. Under low-load conditions, the wear debris consisted mainly of large-area flaky carbon layers (Figure 10a-1). This is primarily because under periodic frictional forces, the carbon layer on the surface of the carbon pin is gradually peeled off, forming flaky debris. Additionally, it also contains lipids that leached from the carbon pin and then condensed. Furthermore, the carbonaceous material encapsulated fragmented strip-like debris (Figure 10a-2). Elemental analysis revealed that this debris was mainly composed of copper (Figure 10a-3,a-4). This indicates that during the current-carrying friction process, the coupling effect of multiple heat sources leads to localized temperature rise on the copper disc surface and subsequent material softening. Under continuous shear stress, the softened copper matrix, after being detached from the substrate, gradually deforms and curls [28], and is eventually cut into coiled strips that become embedded in the wear debris.

When the contact load increased to 50 N, the morphology of the wear debris changed significantly. Under these conditions, the carbon material in the debris appeared mainly as fragmented pieces, while the copper material took on a rod-like shape (Figure 10b-1). This morphological evolution is likely directly related to the increase in contact load. Under high load, the friction pair interface remains in close contact, which not only provides favorable conditions for continuous debris generation but also significantly enhances the shear stress at the interface. The detached copper material undergoes intense curling [29], resulting in a greater quantity and larger volume of copper debris compared with low-load conditions (Figure 10b-2), indicating more severe cutting of the copper disc under high load.

**Figure 11:**
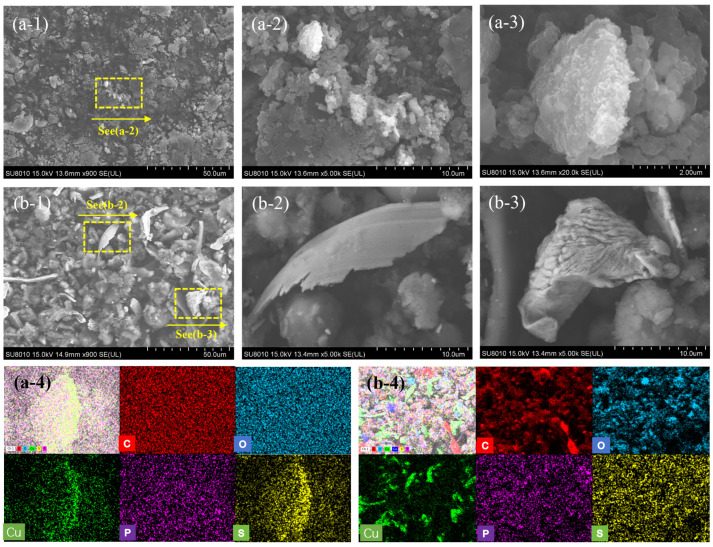
Morphology of wear debris generated by the contact pair under 80 A working condition: (**a-1,a-2,a-3,a-4** ) 25 N; (**b-1,b-2,b-3,b-4**) 50 N.

Figure 11 shows the morphology and elemental distribution of wear debris (category 2 particles) generated by the friction pair during the test at a current intensity of 80 A. At a contact load of 25 N, the wear debris was generally small in size (Figure 11a-1). The carbon material existed in the form of fragmented agglomerates, with a few bright white blocks attached (Figure 11a-2). Elemental analysis revealed that these blocks were composed of Cu and S (Figure 11a-3). Under low-load conditions, arc ablation combined with mechanical action gradually fragmented the carbon material. Meanwhile, the heat released instantaneously by arc discharge caused melting and spattering on the copper disc surface, forming copper vapor. This vapor reacted chemically with sulfur released from the carbon pin ablation at high temperatures [17], generating sulfides that became part of the wear debris. Thus, arc discharge significantly intensifies the chemical erosion of materials during current-carrying friction and wear.

When the contact load increased to 50 N, the morphology and size distribution of the wear debris changed markedly. The flake-like and strip-like debris shown in Figure 11b-1 were both composed of copper (Figure 11b-4), while the blocky debris consisted mainly of carbon material. On the one hand, copper has good ductility. Under a relatively high contact load, the surface asperities peel off under continuous shear stress, forming flake-like and strip-like structures. On the other hand, since the carbon pin is a brittle material, stress tends to concentrate at defects and pores, leading to rapid initiation and propagation of microcracks. Meanwhile, the high current causes a sharp temperature rise on the carbon material surface, generating significant thermal stress between the surface and the interior, which further promotes microcrack initiation and propagation, ultimately causing macroscopic fragments to detach from the bulk material.

**Figure 12:**
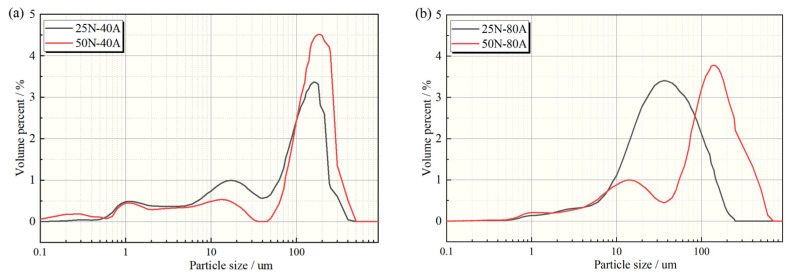
Particle size distribution of wear debris under various current operating conditions: (**a**) 40 A; (**b**) 80 A.

Figure 12 shows the particle size distribution curves of wear debris (category 2 particles) under current-carrying conditions at different contact loads. The wear debris produced under the 0 A condition throughout the entire experiment cycle was so minimal (as illustrated in Figure 5a,d) that particle size analysis was not feasible. Consequently, only the size distributions of the wear debris at 40 A and 80 A are provided in this section. At a current of 40 A, the wear debris size was mostly concentrated around 110 μm (Figure 12a). Compared with the 25 N condition, the volumetric distribution ratio of debris at 10.0 μm decreased significantly under the 50 N condition, while that of debris larger than 100 μm showed an increasing trend. Under high-load conditions, plastic deformation and cutting phenomena were significantly intensified under continuous frictional force. This strong mechanical action combined with thermal effects led to an increased volumetric proportion of large wear debris.

When the current increased to 80 A (Figure 12b), the particle size distribution curve under low-load conditions exhibited a unimodal pattern, with debris sizes mainly concentrated in the range of 10–110 μm. Combined with the wear debris morphology (Figure 11), it is evident that under low-load conditions, intense arc discharge promoted the migration of debris toward smaller sizes, resulting in a homogenized distribution. Under the 50 N condition, however, the synergistic effect of mechanical wear and thermal action broadened the debris size distribution range, showing a multimodal pattern with a significantly increased volumetric proportion of large debris (>100 μm).

## 4. Discussion

**Figure 13:**
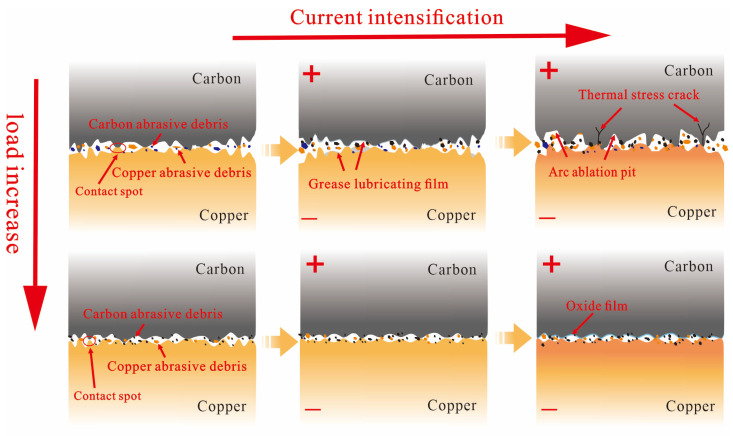
Current-carrying wear mechanism of carbon pin/copper disc under contact load and current.

The wear mechanisms of the contact pair under different contact loads and currents are schematically shown in Figure 13.

Under non-current-carrying conditions, an increase in contact load enlarges the interaction area between asperities on the friction surface and intensifies the cutting action, thereby aggravating mechanical wear. As the friction test proceeds, the surface layer of the friction material is continuously damaged, and increasing amounts of material detach from the friction surface to form particulate matter. Larger particles settle on the copper disc surface, continuing to participate in friction and being crushed. Consequently, the number of fine particles at a contact load of 50 N is higher than that at 25 N.

Under current-carrying conditions, the wear mechanism is jointly influenced by the current magnitude and the contact load. At a current intensity of 40 A and a contact load of 25 N, surface irregularities on the copper disc induce arc discharge at the contact interface. The high temperature released during discharge directly causes the contact surface materials to melt and evaporate, resulting in the formation of a large amount of particulate matter during the initial run-in phase. This not only leads to a sharp increase in particulate emissions but also promotes the formation of a lubricating film on the carbon pin due to heating. This film reduces the interaction between surface asperities in the later stage of friction, mitigating mechanical wear and thus decreasing particulate generation.

When the contact load increases to 50 N, the intensified interaction force between the friction pair aggravates mechanical wear on the friction surface, leading to an increase in spalling pits and severe grooving on the carbon pin surface, which continuously generates particulate matter during the friction process. Overall, although wear intensifies, the particulate matter forms a dynamic buffer layer in the gap of the friction pair, maintaining a relatively stable friction coefficient [30].This is also evidenced by the variation trend of friction coefficients in Figure 2a,b. However, because the total amount of particulate matter generated by mechanical wear increases substantially, the number of particles released into the air per unit time rises and the emission concentration remains stable, resulting in a significantly higher total particle count under this condition compared with the low-load condition.

At a current intensity of 80 A and a contact load of 25 N, intense arc discharge occurs continuously at the friction interface. The enormous heat released instantaneously by the arc discharge rapidly acts on the friction material surface, greatly degrading its structural integrity. Meanwhile, under repeated mechanical impacts, the material surface weakened by high temperatures begins to develop numerous cracks. Along with the formation of many arc erosion craters, the material powders and detaches, making it easier to generate particulate matter during subsequent friction. This results in a persistently high level of particulate emission concentration, with the total number of particles far exceeding that under other conditions. Thus, arc discharge is the main cause of the increase in both the number and concentration of particulate emissions. When the contact load increases to 50 N, the contact area between the friction pair significantly enlarges, resulting in a relatively lower current density on the surface asperities. Under this condition, arc ablation is substantially suppressed. The number concentration of particulate matter emitted by the contact pair begins to decrease after the run-in phase and eventually stabilizes.

## 5. Conclusions

In this paper, a pin-on-disc friction and wear test rig was employed to investigate the effects of varying current intensities under different contact load conditions on the friction and wear performance of carbon/copper pairs and their associated particulate matter emission behavior. It reveals the damage characteristics of carbon contact strips, particulate matter emission properties, and the relationship between them under various service conditions. The wear mechanism and particulate matter emission under current-carrying conditions are jointly influenced by current magnitude and contact load. The main conclusions are as follows:

(1) Under the same contact load, the friction coefficient is highest in the no-current conditions, followed by 80 A, and lowest in 40 A. The wear rate of carbon contact pair shows an upward trend with increasing current. Under different service conditions, the particle size of particulate matter emitted into the air is mainly concentrated at 0.3 μm and 0.5 μm, and the emission concentration of particulate matter at each size increases with increasing current.
(2) When there is no current, as the contact load increases from 25 N to 50 N, the friction coefficient rises significantly. The intensified mechanical action between the friction pair aggravates surface wear, generating more fine particles.
(3) At a contact load of 25 N and a current of 40 A, high-intensity arc discharge in the early stage of friction directly leads to a sharp increase in the number of particles. The damage forms of the carbon pin are mainly grooves and net-like ablation pits. In the later stage of friction, a lubricating film is formed on the surface of the carbon pin, which reduces mechanical wear and arc erosion, thereby suppressing the generation of particles. At a current of 80 A, the surface of the carbon pin is covered with arc ablation craters, electrical wear dominates, and the number of emitted particles increases dramatically.
(4) At a contact load of 50 N, there is no obvious arc ablation on the worn surfaces, and both material damage and particle generation are greatly reduced. Therefore, under the 80 A operating condition, the total number of particles at a contact load of 50 N is approximately one-quarter of that at 25 N, and the volume proportion of large-sized wear debris is also higher than that at 25 N.

Furthermore, the limitations of this study are explained as follows. Due to the complexity of the pantograph–catenary system operation, it is very difficult to fully replicate actual contact conditions in a laboratory environment. Therefore, in this study, a pin-on-disk tribometer was used to simulate the current-carrying friction between the carbon strip and the contact wire, which is a common approach in this field (e.g., References [31,32,33]). Nevertheless, it must be acknowledged that there remains a considerable gap between the experimental setup and parameter settings of an actual pantograph–catenary system, including parameters such as the stagger, operating speed, and electric current during operation. Further research aimed at improving the consistency between the testing equipment and real-world operating conditions is still needed.

## Figures and Tables

**Table 1:** The chemical compositions of the two components of the friction pair.

Samples	Chemical Component (wt./%)					
Carbon pin	C	O	S/P/Cl	-	-	-
	84.85	10.2	bal.	-	-	-
Cu disc	Cu	O	Fe	S	P	Ni/Sb/Pb
	99.95	0.006	0.005	0.005	0.008	bal.

**Table 2:** Test parameters for different current tests under two types of contact loads.

Test Conditions	Parameter Value
Contact load *F_n_* (N)	25, 50
Rotating speed of copper disc *v* (Hz)	1
Radius of friction *R* (mm)	82
Test current *I* (A)	0, 40, 80
Test time *T* (s)	1800

## Data Availability

The original contributions presented in this study are included in the article. Further inquiries can be directed to the corresponding author.

## References

[1] Bendl J., Neukirchen C., Mudan A., Padoan S., Zimmermann R., Adam T. (2023). Personal Measurements and Sampling of Particulate Matter in a Subway—Identification of Hot-Spots, Spatio-Temporal Variability and Sources of Pollutants. Atmos. Environ..

[2] Wang X., Meng X., Chang L., Pei F., Wan T., Cui T., Liu Y., Pan S. (2024). Concentration, Composition and Exposure Risk Assessment of Particulate Matter with Different Particle Sizes in Subway Stations. Build. Environ..

[3] Zhu Y., Kan H. (2025). Current Research Status and Advances in the Impact of Atmospheric Environment on Human Health. J. Lanzhou Univ. Med. Sci..

[4] Chen H., Yan Y., Hu D., Peng L., Wang C. (2024). PM2.5-Bound Heavy Metals in a Typical Industrial City of Changzhi in North China: Pollution Sources and Health Risk Assessment. Atmos. Environ..

[5] Wang D., Shen H. (2019). Analysis of Air Quality in Shanghai Subway Station. Build. Energy Environ..

[6] Karlsson H.L., Nilsson L., Möller L. (2005). Subway Particles Are More Genotoxic than Street Particles and Induce Oxidative Stress in Cultured Human Lung Cells. Chem. Res. Toxicol..

[7] Zhang Y., Chu M., Zhang J., Duan J., Hu D., Zhang W., Yang X., Jia X., Deng F., Sun Z. (2019). Urine Metabolites Associated with Cardiovascular Effects from Exposure of Size-Fractioned Particulate Matter in a Subway Environment: A Randomized Crossover Study. Environ. Int..

[8] Huang S., Chen P., Hu K., Qiu Y., Feng W., Ren Z., Wang X., Huang T., Wu D. (2021). Characteristics and Source Identification of Fine Particles in the Nanchang Subway, China. Build. Environ..

[9] Colombi C., Angius S., Gianelle V., Lazzarini M. (2013). Particulate Matter Concentrations, Physical Characteristics and Elemental Composition in the Milan Underground Transport System. Atmos. Environ..

[10] Martins V., Moreno T., Mendes L., Eleftheriadis K., Diapouli E., Alves C.A., Duarte M., De Miguel E., Capdevila M., Querol X. (2016). Factors Controlling Air Quality in Different European Subway Systems. Environ. Res..

[11] Fruhwirt D., Sturm P., Bucca G., Bode G., Michael S., Rodler J. (2023). Emissions from Railways: Results of Tests on a Pantograph-Catenary Test Bench. Transp. Res. Part Transp. Environ..

[12] Sunar O., Fletcher D., Beagles A. (2021). Laboratory Assessment of ARC Damage in Railway Overhead Contact Lines With a Case Study on Copper-Silver and Low Oxygen Content Copper. IEEE Trans. Power Deliv..

[13] Mei G., Fu W., Chen G., Zhang W. (2020). Effect of High-Density Current on the Wear of Carbon Sliders against Cu–Ag Wires. Wear.

[14] Zhang Y.Y., Zhang Y.Z., Du S.M., Song C.F., Yang Z.H., Shangguan B. (2018). Tribological Properties of Pure Carbon Strip Affected by Dynamic Contact Force during Current-Carrying Sliding. Tribol. Int..

[15] Zhang Y., Li C., Pang X., Song C., Ni F., Zhang Y. (2021). Evolution Processes of the Tribological Properties in Pantograph/Catenary System Affected by Dynamic Contact Force during Current-Carrying Sliding. Wear.

[16] Yang H.J., Chen G.X., Gao G.Q., Wu G.N., Zhang W.H. (2015). Experimental Research on the Friction and Wear Properties of a Contact Strip of a Pantograph–Catenary System at the Sliding Speed of 350 Km/h with Electric Current. Wear.

[17] Font O., Moreno T., Querol X., Martins V., Sánchez Rodas D., De Miguel E., Capdevila M. (2019). Origin and Speciation of Major and Trace PM Elements in the Barcelona Subway System. Transp. Res. Part Transp. Environ..

[18] Mei G. (2020). Tribological Performance of Rigid Overhead Lines against Pantograph Sliders under DC Passage. Tribol. Int..

[19] Hu Y., Huang P., Cheng C., Zhang M., Ma R. (2024). Influence of Arc Discharge on the Temperature and Wear Behaviors of the Contact Strip in Pantograph-Rigid Catenary Systems under AC Conditions. Wear.

[20] Liu X., Zheng Y., Deng G., Xiao Q., Shen M., Zhang D., Cao H., Wang Z., Gao M., Wu H. (2024). Comparative Study of the Current-Carrying Property of Different Copper-Impregnated Carbon Skateboards/Contact Wires. Tribol. Int..

[21] Huang M., Yang B., Rong Y., Zhou G.D., Li J.L., Wang S.Q. (2023). Study on Friction and Wear Properties of Copper-Impregnated Carbon Slide Plate Under Different Humidity Conditions. Tribol. Trans..

[22] Deng C., Yin J., Zhang H., Xiong X., Wang P., Sun M., Wu X. (2019). Dynamic Variation of Arc Discharge and Its Effect on Corrosion Direction under Current-Carrying Sliding. Proc. Inst. Mech. Eng. Part J J. Eng. Tribol..

[23] Zhou Y., Du M., Zuo X. (2022). Influence of Electric Current on the Temperature Rise and Wear Mechanism of Copper–Graphite Current-Carrying Friction Pair. J. Tribol..

[24] Jin M., Hu M., Li H., Yang Y., Liu W., Fang Q., Liu S. (2022). Experimental Study on the Transient Disturbance Characteristics and Influence Factors of Pantograph–Catenary Discharge. Energies.

[25] Li K., Wang X., Jiang L., Rajurkar K.P., Zhao W., Gu L. (2024). Study of Arc Behavior and Machining Effects of the Novel Magnetic Field Assisted Blasting Erosion Arc Machining Method. J. Mater. Process. Technol..

[26] Luo X., Cai C., Yang H., Mei G., Gao C., Liu W., Yang D. (2023). Experimental Research on the Non-Uniform Wear of the Carbon Strip of the Metro Pantograph. Proc. Inst. Mech. Eng. Part J J. Eng. Tribol..

[27] Shen M., Ji D., Hu Q., Xiao L., Li Q. (2024). Current-Carrying Tribological Behavior of C/Cu Contact Pairs in Extreme Temperature and Humidity Environments for Railway Catenary Systems. Sci. China Technol. Sci..

[28] Shu X., Liu Y., Li W. (2022). Research Progress in Current-Carrying Friction and Wear of Copper Matrix Composites. Spec. Cast. Nonferrous Alloys.

[29] Yang Z., Song Y., Jiao J., Li W., Shangguan B., Zhang Y. (2024). Optimization of Current-Carrying Friction and Wear Properties of Copper-Carbon Composite Materials Based on Damage. Tribol. Int..

[30] Kim J.W., Joo B.S., Jang H. (2019). The Effect of Contact Area on Velocity Weakening of the Friction Coefficient and Friction Instability: A Case Study on Brake Friction Materials. Tribol. Int..

[31] Ding T., Chen G.X., Zhu M.H., Zhang W.H., Zhou Z.R. (2009). Influence of the Spring Stiffness on Friction and Wear Behaviours of Stainless Steel/copper-impregnated Metallized Carbon Couple with Electrical Current. Wear.

[32] Zhang Y., Zhang Y., Song C. (2018). Arc Discharges of a Pure Carbon Strip Affected by Dynamic Contact Force During Current-Carrying Sliding. Materials.

[33] Ding T., Chen G.X., Li Y.M., He Q., Xuan W. (2012). Friction and Wear Behavior of Pantograph Strips Sliding Against Copper Contact Wire with Electric Current. AASRI Procedia.

